# Baseline MRI Predictors of Conversion from MCI to Probable AD in the ADNI Cohort

**DOI:** 10.2174/156720509788929273

**Published:** 2009-08

**Authors:** Shannon L Risacher, Andrew J Saykin, John D West, Li Shen, Hiram A Firpi, Brenna C McDonald, the Alzheimer’s Disease Neuroimaging Initiative (ADNI)

**Affiliations:** 1 IU Center for Neuroimaging, Division of Imaging Sciences, Department of Radiology, Indiana University School of Medicine, 950 W Walnut St, R2 E124, Indianapolis, IN 46202, USA; 2 Medical Neuroscience Program, Stark Neurosciences Research Institute, Indiana University School of Medicine, 950 W Walnut St, R2 Building, Room 402, Indianapolis, IN 46202, USA; 3 Indiana Alzheimer Disease Center, Indiana University School of Medicine, Indianapolis, IN 46202, USA; 4 Center for Computational Biology and Bioinformatics, Indiana University School of Medicine, 410 West 10th Street, Suite 5000, Indianapolis, IN 46202, USA

**Keywords:** Alzheimer’s disease neuroimaging initiative (ADNI), magnetic resonance imaging (MRI), mild cognitive impairment (MCI), hippocampus, cognition.

## Abstract

The Alzheimer’s Disease Neuroimaging Initiative (ADNI) is a multi-center study assessing neuroimaging in diagnosis and longitudinal monitoring. Amnestic Mild Cognitive Impairment (MCI) often represents a prodromal form of dementia, conferring a 10-15% annual risk of converting to probable AD. We analyzed baseline 1.5T MRI scans in 693 participants from the ADNI cohort divided into four groups by baseline diagnosis and one year MCI to probable AD conversion status to identify neuroimaging phenotypes associated with MCI and AD and potential predictive markers of imminent conversion. MP-RAGE scans were analyzed using publicly available voxel-based morphometry (VBM) and automated parcellation methods. Measures included global and hippocampal grey matter (GM) density, hippocampal and amygdalar volumes, and cortical thickness values from entorhinal cortex and other temporal and parietal lobe regions. The overall pattern of structural MRI changes in MCI (n=339) and AD (n=148) compared to healthy controls (HC, n=206) was similar to prior findings in smaller samples. MCI-Converters (n=62) demonstrated a very similar pattern of atrophic changes to the AD group up to a year before meeting clinical criteria for AD. Finally, a comparison of effect sizes for contrasts between the MCI-Converters and MCI-Stable (n=277) groups on MRI metrics indicated that degree of neurodegeneration of medial temporal structures was the best antecedent MRI marker of imminent conversion, with decreased hippocampal volume (left > right) being the most robust. Validation of imaging biomarkers is important as they can help enrich clinical trials of disease modifying agents by identifying individuals at highest risk for progression to AD.

## INTRODUCTION

Alzheimer’s disease (AD) is the most common neurodegenerative illness associated with aging, accounting for 60-70% of age-related dementia cases. In 2000, approximately 25 million people over the age of 60 were diagnosed with dementia worldwide, and the number afflicted is expected to reach over 80 million by 2040 [1, 2]. Earlier diagnosis of AD is widely considered to be an important goal for researchers. Characterization of the earliest known clinical signs has led to the development of the classification of Mild Cognitive Impairment (MCI), which is thought to be a transitional stage between normal aging and the development of AD [3]. Patients with MCI, specifically those with primary memory deficits or “amnestic MCI”, have a significantly higher likelihood to progress to probable AD, with a conversion rate of 10-15% per year [4]. Therefore, MCI represents an important clinical group in which to study longitudinal changes associated with the development of AD. The detection of subtle changes in brain structure associated with disease progression and the development of tools to detect those who are most likely to convert from MCI to probable AD is an important goal.

The Alzheimer's Disease Neuroimaging Initiative (ADNI) is a five-year public-private partnership to test whether serial magnetic resonance imaging (MRI), positron emission tomography (PET), other biological markers, and clinical and neuropsychological assessment can be combined to measure the progression of amnestic MCI and early probable AD [5-7]. One of the major goals of ADNI is to assess selected neuroimaging and analysis techniques for sensitivity and specificity for both cross-sectional diagnostic group classification and longitudinal progression of MCI and AD.

A powerful technique for analyzing high resolution structural MRI data is voxel-based morphometry (VBM), which allows specific tissue classes (i.e., grey matter (GM), white matter (WM), or CSF) to be analyzed in an automated and unbiased manner [8-10]. VBM analyses, particularly comparisons of GM density between groups, have been used to examine diagnostic group differences in both cross-sectional and longitudinal studies of brain aging and AD [11-30]. In fact, VBM has been shown to accurately classify controls and AD patients and to predict conversion from MCI to AD and rate of progression in studies of brain aging [12, 13, 15, 18, 20, 23]. However, the small sample size of these studies and minimal longitudinal monitoring has prevented VBM from being established as a conclusive biomarker for MCI to probable AD conversion.

Regions of interest (ROIs) and volumes of interest (VOIs) have also been effective in measuring local atrophy associated with AD and MCI and longitudinal monitoring of neurodegeneration in studies of brain aging. Numerous studies using manually defined ROIs have found that local hippocampal and total brain volume are significantly reduced in AD and MCI patients relative to healthy elderly individuals [17, 24, 25, 28, 31-46]. Rates and amount of hippocampal, medial temporal lobe (MTL), and total brain atrophy have also been shown to correlate with MCI to AD conversion [31-34, 37, 40-42, 44, 45, 47-52]. Recently, automated methods for extraction of specific regional volumes have been developed and found to provide similar reliability as manually traced ROIs in AD [53-56]. Automated parcellation methods have also demonstrated reliable cortical thickness value estimations and decreased cortical thickness in AD [56, 57].

The goal of the present study was to perform group comparisons using the 1.5T T1-weighted structural scans obtained from ADNI participants at baseline. Using VBM as implemented in SPM5 (http://www.fil.ion.ucl.ac.uk/spm/), we examined cross-sectional GM differences between groups stratified by baseline diagnosis and one year conversion from MCI to probable AD. Study groups included participants diagnosed with AD at the screening, baseline, 6-, and 12-month follow-up visits (AD), participants designated as healthy elderly controls at all four visits (HC), participants who were diagnosed with MCI at all four visits (MCI-Stable), and participants who were diagnosed with MCI at baseline and converted from MCI to probable AD within the first year (MCI-Converters). We extracted bilateral hippocampal GM density values, hippocampal and amygdalar volumes, and entorhinal cortex, temporal lobe, and parietal lobe cortical thickness values for between-group comparisons. We hypothesized that patients with AD would show extensive GM reduction in medial and lateral temporal lobes and other neocortical regions, and that both of the MCI groups would demonstrate focal reduction in MTL structures compared to HC. We also hypothesized that MCI participants who converted to AD within one year would show a more extensive pattern of global GM reduction relative to HC, particularly in regions of the MTL, than participants with a stable diagnosis of MCI, but a less extensive pattern than AD participants. We predicted that MCI-Converters would show greater MTL and neocortical GM density reductions relative to MCI-Stable participants. Finally, we investigated whether local hippocampal GM density and volume, amygdalar volume, and entorhinal, temporal, and parietal cortical thickness values would reflect the same pattern of group differences, and the relative ability of these MRI metrics to detect differences between MCI-Converter and MCI-Stable groups.

## METHODS

### ADNI

ADNI was launched in 2004 by the National Institute on Aging (NIA), the National Institute of Biomedical Imaging and Bioengineering (NIBIB), the Food and Drug Administration (FDA), private pharmaceutical companies, and non-profit organizations. More than 800 participants, ages 55-90, have been recruited from 59 sites across the U.S. and Canada to be followed for 2-3 years. The primary goal of ADNI is to determine whether serial magnetic resonance imaging (MRI), positron emission tomography (PET), other biological markers, and clinical and neuropsychological assessment can accurately measure the progression of MCI and early AD. The identification of specific biomarkers of early AD and disease progression will provide a useful tool for researchers and clinicians in both the diagnosis of early AD and in the development, assessment and monitoring of new treatments. For additional information about ADNI, see www.adni-info.org.

### MRI Scans

Baseline 1.5T MRI scans from 820 participants were downloaded from the ADNI public website (http://www.loni. ucla.edu/ADNI/) onto local servers at Indiana University School of Medicine between January and April 2008. The downloaded data initially included baseline scans from 229 HC, 403 patients with MCI, and 188 patients with AD. Complete details regarding participant exclusion and categorization are provided in Fig. (**1**). Scan data was acquired on 1.5T GE, Philips, and Siemens MRI scanners using a magnetization prepared rapid acquisition gradient echo (MP-RAGE) sequence that was selected and tested by the MRI Core of the ADNI consortium [5]. Briefly, two high-resolution T1-weighted MRI scans were collected for each participant using a sagittal 3D MP-RAGE sequence with an approximate TR=2400ms, minimum full TE, approximate TI=1000ms, and approximate flip angle of 8 degrees (scan parameters vary between sites, scanner platforms, and software versions). Scans were collected with a 24cm field of view and an acquisition matrix of 192 x 192 x 166 (x, y, z dimensions), to yield a standard voxel size of 1.25 x 1.25 x 1.2 mm. Images were then reconstructed to give a 256 x 256 x 166 matrix and voxel size of approximately 1 x 1 x 1.2 mm. Additional scans included prescan and scout sequences as indicated by scanner manufacturer, axial proton density T2 dual contrast FSE/TSE, and sagittal B1-calibration scans as needed. Further details regarding the scan protocol can be found in [5] and at www.adni-info.org. Scans were collected at either screening (n=845) or baseline visits (n=184) between August 2005 and October 2007. If scans existed from both sessions for a single participant, the scan from the screening visit was used. Details of the ADNI design, participant recruitment, clinical testing, and imaging methods, have been published previously [5, 6, 7, http://www.adni-info.org/].

### Image Processing

VBM: Analysis was performed using previously described methods [8-10], as implemented in SPM5 (http://www. fil.ion.ucl.ac.uk/spm/). Briefly, scans were converted from DICOM to NIfTI format, co-registered to a standard T1 template image, bias corrected, and segmented into GM, WM, and CSF compartments using standard SPM5 templates. GM maps were then normalized to MNI atlas space as 1x1x1 mm voxels and smoothed using a 10 mm FWHM Gaussian kernel. In cases where the first MP-RAGE scan could not be successfully segmented we attempted to use the second MP-RAGE. This was successful for only 1 of 8 cases.

Region of Interest: A hippocampal ROI template was created by manual tracing of the left and right hippocampi in an independent sample of 40 HC participants enrolled in our study of brain aging and MCI at Dartmouth Medical School [25, 58]. These ROIs were used to extract GM density values from smoothed, unmodulated normalized and modulated normalized GM maps for the ADNI cohort.

Automated Parcellation: VOIs, including bilateral hippocampi and amygdalar nuclei, were extracted using FreeSurfer V4 [56, 59-62]. FreeSurfer was also used to extract cortical thickness values from the left and right entorhinal cortex, inferior, middle, and superior temporal gyri, inferior parietal gyrus, and precuneus.

**Fig. (1):**
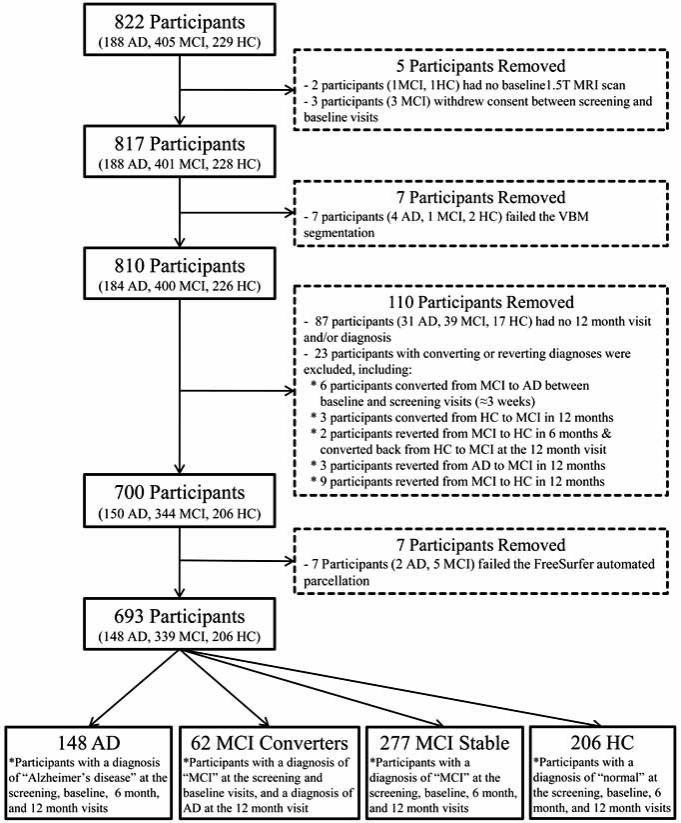
Flowchart of participant pool selection with group exclusion and inclusion criteria.

The final sample reported here passed site, ADNI MRI Core, and our internal quality control, and did not fail any step of the processing pipeline (Fig. **1**).

### Demographic Data

Demographic information, ApoE genotype, neuropsychological test scores, and diagnosis were downloaded from the ADNI clinical data repository (https://www.loni.ucla.edu/ ADNI/Data/ADCS_Download.jsp). The “10-27-08” version of the ADNI clinical database was used for all analyses. Participants were initially classified into groups based on screening or baseline diagnosis as reported in the diagnosis and conversion/reversion database.

### VBM Statistical Analyses

Statistical analyses were performed on a voxel-by-voxel basis using a general linear model (GLM) approach implemented in SPM5. A false discovery rate (FDR) adjustment was used where appropriate to control for multiple comparisons, and a minimum cluster size (k) of 27 voxels was required for significance. Age, gender, years of education, handedness, and total intracranial volume (ICV) were included as covariates, and an explicit GM mask was used to restrict analyses to GM regions. A one-way ANOVA was performed to compare the smoothed, unmodulated normalized GM maps between groups to determine the effects of diagnosis and one year conversion from MCI to AD on GM density. The initial comparison was done using the entire available sample of 693 participants. A second comparison was completed using the same methods but with subgroups of matched participants to correct for unequal group sizes (n=248; 62 in each group). Matching was done on a case by case basis using the best available match on age, gender, education, and handedness, while preserving the relative proportion of ApoE4+ participants within each subgroup. After matching there were no significant group differences in age, gender, education, or handedness.

Finally, a third set of analyses were performed with the full available sample of 693 participants, adding a volume preserving modulation step to the VBM method, yielding an assessment of local GM volume differences instead of GM density.

### Other Statistical Analyses

Mean left and right hippocampal GM density, hippocampal and amygdalar volumes, and cortical thickness values for all 693 participants were compared between groups using a one-way multivariate ANOVA in SPSS (version 16.0.1). Age, gender, education, handedness, and ICV were included as covariates in all ROI, VOI, and cortical thickness comparisons. One-way ANOVA and chi-square tests were used to determine between-group differences in age, gender distribution, ApoE genotype, education, handedness distribution, primary language distribution, and baseline global, functional, behavioral, neurological, neuropsychiatric, and neuropsychological test scores. All graphs were created using SigmaPlot (version 10.0).

Effect sizes for the comparison between MCI-Converters and MCI-Stable participants were also calculated for selected imaging biomarkers, including bilateral hippocampal GM density and volume from the VBM images, bilateral hippocampal, amygdalar, accumbens, ventral dorsal column, inferior lateral ventricle, lateral ventricle, cerebral cortex, and cerebral white matter volumes extracted using automated parcellation, and cortical thickness values from bilateral entorhinal cortex, inferior, middle, and superior temporal gyri, inferior parietal gyrus, and precuneus, which were also extracted using automated parcellation. These values were assessed due to significant differences between MCI-Converter and MCI-Stable groups on pairwise comparisons (p<0.05). Left and right adjusted means for each imaging measure, covaried for age, gender, education, handedness, and ICV, were averaged to give a bilateral estimate, and used to calculate effect sizes (Cohen’s d) in SPSS and Microsoft Excel (version 2007).

## RESULTS

### Sample Characteristics

Demographic information and mean baseline test scores for all groups are presented in Table **1**. Mean participant age and handedness distribution did not differ across groups. Years of education and percent of participants with either one or two ApoE ε4 alleles (ApoE4+) were significantly different between diagnosis groups (p<0.001). AD participants showed significantly fewer years of education than either HC (p<0.001) or MCI-Stable (p=0.003) participants. Years of education did not differ significantly between any other groups in pairwise comparisons. As expected, the HC group had a lower percentage of ApoE4+ participants than any of the clinical groups, while the AD group had the highest percentage of ApoE4+ participants. The MCI-Stable and MCI-Converter groups had different proportions of ApoE4+ participants, with the MCI-Converter group showing a higher percentage of ApoE4+ participants than the MCI-Stable group. Neuropsychiatric test results, including scores from the Geriatric Depression Scale (GDS) [63] and Neuropsychiatric Inventory Questionnaire (NPI-Q) [64], were significantly different between groups (p<0.001). HC participants showed significantly fewer depressive symptoms than either AD or MCI-Stable participants (p<0.001), and had a lower mean score on the NPI-Q than the AD, MCI-Stable, and MCI-Converter groups (p<0.001). AD participants also had a significantly higher mean NPI-Q score than the MCI-Stable (p<0.001) and MCI-Converter (p=0.008) groups. No significant differences in mean GDS scores were found between the MCI-Stable, MCI-Converter, and AD groups. No group showed clinically meaningful levels of depressive symptoms. Ischemic events and/or risk were not significantly different between groups as assessed by the Modified Hachinski scale [65].

As expected, neuropsychological test scores (MMSE [66, 67], Clinical Dementia Rating (CDR) [68], and the Functional Assessment Questionnaire (FAQ) [69]) varied significantly between groups (p<0.001). Pairwise comparisons showed a similar pattern for the MMSE, Global CDR, and CDR-Sum of Boxes. HC participants had significantly higher MMSE and lower CDR scores relative to all other groups (p<0.001). Additionally, MCI-Stable and MCI-Converter participants showed significantly higher MMSE and lower CDR scores compared to AD participants (p<0.001), but did not differ from one another on these assessments. Mean FAQ total scores were significantly different across groups and in all pairwise comparisons (p<0.001).

Neuropsychological scores from the Rey Auditory Verbal Learning Test (RAVLT) [70], Boston Naming Test (BNT) [71], and category verbal fluency tests (Fluency-Animals, Fluency-Vegetables) [72] also showed significant differences between groups (p<0.001). However, these assessments showed a different pattern in pairwise comparisons than the MMSE, Global CDR, and FAQ. MCI-Converters and AD participants showed similar scores on the learning and verbal neuropsychological tests, with no significant differences on RAVLT measures, BNT, or verbal fluency tests. As expected, all of the clinical groups performed below HC participants for RAVLT, BNT, and verbal fluency measures (p<0.001). MCI-Stable participants also had significantly higher scores on all RAVLT measures and Fluency-Vegetables than both the AD and MCI-Converter groups (p<0.001). Finally, MCI-Stable participants had significantly higher scores than AD participants but not MCI-Converters on Fluency-Animals and BNT (p<0.001).

### VBM Group Comparisons by Baseline Diagnosis and One Year Conversion Status

**Fig. (2). Group comparisons of healthy control participants and patient groups using a one-way ANOVA of GM density maps.**
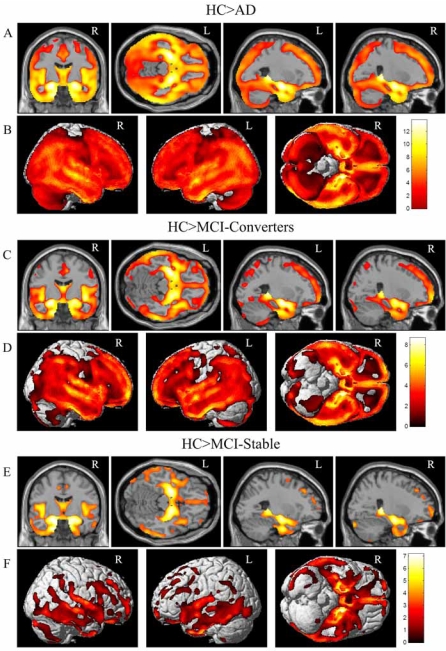
Selected slices (**A**) and surface renderings (**B**) of regions where HC>AD. Selected slices (**C**) and surface renderings (**D**) of regions where HC>MCI-Converters. Selected slices (**E**) and surface renderings (**F**) of regions where HC>MCI-Stable. All comparisons are displayed at a threshold of p<0.005 (FDR), k=27. Age, gender, years of education, handedness and ICV were included as covariates in all comparisons. Selected sections for (**A**), (**C**), and (**E**) include left to right MNI coordinates: (0, -9, 0, coronal), (0, -23, -16, axial), (-26, -10, -15, sagittal), and (26, -10, -15, sagittal).

All 693 participants were included in the initial VBM analyses. A one-way ANOVA indicated striking between-group differences in smoothed, unmodulated normalized GM maps (see Figs. **2 & 3**, unless noted, all differences are p<0.005 (FDR)). AD participants showed reduced density in nearly all GM regions compared to the HC group, with the maximum global difference in the left hippocampus (Fig. **2a**, HC>AD). Surface renderings of the comparison between the HC and AD groups showed that the GM density of nearly the entire cortical surface is significantly lower in AD (Fig. **2b**, HC>AD), including significant differences in the temporal, frontal and parietal lobes. MCI-Converters also showed reduced GM density compared to HC, with a global maximum in the left hippocampus (Fig. **2c**, HC>MCI-Converters). The pattern of significant voxels in the comparison between HC and MCI-Converters was very similar to that seen in the HC>AD comparison, both in subcortical regions and on the cortical surface (Fig. **2d**, HC>MCI-Converters). Selected sections (Fig. **2e**, HC>MCI-Stable) show a more focal distribution of differences in the comparison of GM maps from MCI-Stable and HC participants. MCI-Stable participants showed reduced GM density in focal bilateral MTL regions relative to HC, with a global maximum in the right parahippocampal gyrus and additional local maxima in bilateral amygdalar and hippocampal regions. Surface renderings reflect the focal distribution pattern of significant voxels in the HC>MCI-Stable contrast (Fig. **2f**, HC>MCI-Stable), with differences localized primarily in the temporal and frontal lobes.

A widespread pattern of significant voxels was also detected in the comparison between the MCI-Stable and AD groups. MCI-Stable participants showed significantly higher GM density than AD in the MTL, including a global maximum difference in the left hippocampus (Fig. **3a**, MCI-Stable>AD) and additional local maxima in bilateral amygdalar and hippocampal regions. The extensive pattern of GM differences between MCI-Stable and AD participants is further reflected in the surface renderings, with AD participants having significant GM reduction on nearly the entire cortical surface relative to MCI-Stable participants (Fig. **3b**, MCI-Stable>AD). A more focal pattern was observed when comparing MCI participant groups. MCI-Converters had significantly reduced GM density relative to MCI-Stable participants in bilateral MTL regions, with a global maximum in the right insula and additional local maxima in bilateral amygdalar and hippocampal regions (Fig. **3c**, MCI-Stable>MCI-Converters). Surface renderings of the comparison between the MCI-Stable and MCI-Converter groups also show a focal pattern of GM differences in the frontal and temporal lobes (Fig. **3d**, MCI-Stable>MCI-Converters). No significant voxels were found in the comparison between GM density maps from MCI-Converters and AD participants (Fig. **3**, MCI-Converters>AD). At a lower statistical threshold (p<0.001 (uncorrected)), AD participants showed reduced GM density in focal regions of the posterior parietal and occipital lobes relative to MCI-Converters (data not shown).

As noted above, similar contrasts were performed using matched participants in equal sized groups to control for power as a function of group size. This comparison resulted in a highly similar pattern of between-group differences as in the full sample but, as anticipated, at lower statistical thresholds due to attenuated power (data not shown). Results from comparisons between groups using modulated normalized GM maps from the full sample were also highly similar to those using unmodulated images (data not shown).

### ROI Grey Matter Density Comparisons

**Fig. (4). Extracted GM density, volume, and cortical thickness values from medial temporal lobe structures.**
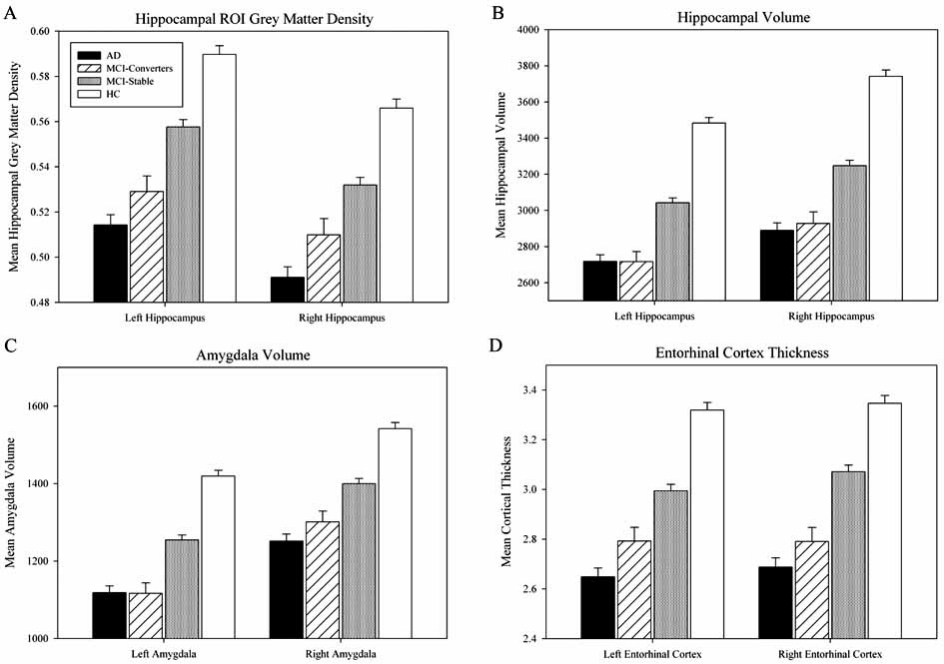
Comparisons of GM density values (**A**) were extracted from the unmodulated VBM GM maps using standard left and right hippocampal ROIs traced on an independent sample of 40 HC participants [25, 58]. Bilateral hippocampal (**B**) and amygdalar (**C**) volume estimates and entorhinal cortex thickness values (**D**) were extracted using automated parcellation. The comparisons of all four MRI metrics show a significant difference (p<0.001) across all groups. In pairwise comparisons, hippocampal GM density, hippocampal and amygdala volumes, and entorhinal cortex thickness show significant differences between HC and all clinical groups (p<0.001) bilaterally and MCI-Stable and AD groups (p<0.001) bilaterally. Furthermore, MCI-Stable and MCI-Converter groups show significant differences in GM density and volume in the left (GM (**A**), p=0.001; volume (**B**), p<0.001) and right (GM (**A**), p=0.034; volume (**B**), p<0.001) hippocampi, as well as significant differences in amygdala volume on both the left (p<0.001) and right (p=0.01). MCI-Converters also showed significantly thinner entorhinal cortices than MCI-Stable participants on both the left (p=0.006) and right (p<0.001). No significant differences were found in hippocampal GM density, hippocampal or amygdalar volumes, or entorhinal cortex thickness values between MCI-Converter and AD groups. Age, gender, years of education, handedness, and ICV were included as covariates in all comparisons.

Mean left and right hippocampal GM density values from the smoothed, unmodulated normalized GM maps of all 693 participants were extracted as described above. GM density was significantly different between all groups for both the left and right hippocampi (Fig. **4a**, p<0.001). In pairwise comparisons, HC participants showed significantly greater hippocampal GM density bilaterally relative to all other groups (p<0.001). MCI-Converters had significantly reduced local GM density relative to MCI-Stable participants in both the left (p=0.001) and right (p=0.034) hippocampi, as did AD participants (p<0.001 bilaterally). Hippocampal GM density did not differ significantly between AD participants and MCI-Converters. Analyses using smoothed, modulated normalized GM maps showed a similar pattern of results to those using unmodulated images (data not shown).

### FreeSurfer-Derived VOI and Cortical Thickness Comparisons

Bilateral hippocampal and amygdalar volumes and cortical thickness values from the entorhinal cortex, inferior, middle, and superior temporal gyri, inferior parietal gyrus and precuneus were extracted from all 693 participants as described above. Comparisons of mean bilateral hippocampal (Fig. **4b**) and amygdalar (Fig. **4c**) volumes and entorhinal cortex thickness (Fig. **4d**) were significant across all groups (p<0.001), and show similar results in pairwise comparisons. All of the clinical groups (MCI-Stable, MCI-Converters, AD) had decreased bilateral hippocampal and amygdalar volumes and entorhinal cortex thickness compared to HC (p<0.001). MCI-Converters also showed significant reductions relative to MCI-Stable participants, including reduced bilateral hippocampal volumes (p<0.001), bilateral amygdalar volumes (p<0.001 left, p=0.01 right), and thinner bilateral entorhinal cortex (p=0.006 left, p<0.001 right). AD participants had significant reductions in bilateral hippocampal and amygdalar volumes and entorhinal cortex thickness relative to MCI-Stable participants (p<0.001). However, MCI-Converters and AD participants showed no significant differences in any MTL measures (hippocampal and amygdalar volume or entorhinal cortex thickness).

**Fig. (5). Cortical thickness values from the temporal lobe extracted using automated parcellation.**
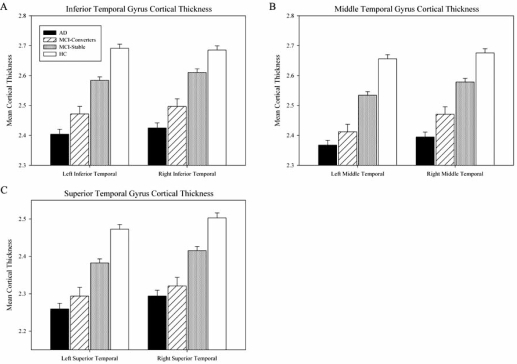
Comparisons between cortical thickness values from three regions of the temporal lobe, including inferior (**A**), middle (**B**), and superior (**C**) temporal gyri, demonstrated significant differences (p<0.001) across all groups. Pairwise comparisons demonstrated significant differences in cortical thickness values for all temporal gyri bilaterally between HC and all other groups (p<0.001), as well as between the MCI-Stable and AD groups (p<0.001). The MCI-Converter and MCI-Stable groups also showed significant differences in cortical thickness in bilateral inferior temporal gyri (p<0.001), left (p<0.001) and right (p=0.001) middle temporal gyri, and left (p=0.003) and right (p=0.002) superior temporal gyri. Cortical thickness values from bilateral inferior, middle, and superior temporal gyri were not significantly different between the MCI-Converter and AD groups. Age, gender, years of education, handedness, and ICV were included as covariates in all comparisons.

Mean cortical thickness values from lateral temporal cortices were also significantly different across groups (Fig. **5**, p<0.001). Similar to other ROI and VOI comparisons, HC participants had significantly greater bilateral inferior (Fig. **5a**), middle (Fig. **5b**) and superior (Fig. **5c**) temporal gyrus cortical thickness relative to all other groups in pairwise comparisons (p<0.001). MCI-Converters had significant cortical thinning bilaterally relative to MCI-Stable participants in the inferior (p<0.001), middle (p<0.001 left, p=0.001 right), and superior (p=0.003 left, p=0.002 right) temporal gyri. AD participants also had significantly thinner bilateral inferior, middle, and superior temporal gyri relative to MCI-Stable participants (p<0.001). MCI-Converters and AD participants showed no temporal gyrus cortical thickness differences.

**Fig. (6). Parietal cortical thickness values extracted using automated parcellation.**
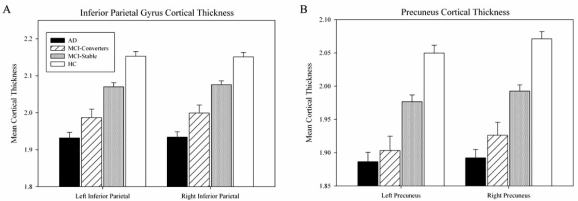
Cortical thickness values from the inferior parietal gyrus (**A**) and precuneus (**B**) showed significant differences between groups (p<0.001). Pairwise comparisons showed significant differences in bilateral inferior parietal gyrus and precuneus between HC and all clinical groups (p<0.001), as well as between the MCI-Stable and AD groups (p<0.001). The MCI-Stable and MCI-Converter groups were significantly different in the left (p=0.006) and right (p=0.009) inferior parietal gyri and left (p=0.012) and right (p=0.013) precuneus. No significant difference was found between MCI-Converter and AD groups in either region. Age, gender, years of education, handedness and ICV were included as covariates in all comparisons.

Parietal lobe cortical thickness values also showed significant differences across all groups, specifically in the inferior parietal gyrus (Fig. **6a**, p<0.001) and precuneus (Fig. **6b**, p<0.001). Pairwise comparisons showed similar patterns as those of other imaging biomarkers. HC participants had significantly greater cortical thickness in bilateral inferior parietal gyrus and precuneus relative to all other groups (p<0.001). AD participants had significantly reduced cortical thickness in bilateral inferior parietal and precuneus regions relative to MCI-Stable participants (p<0.001), as did MCI-Converters (inferior parietal gyrus p=0.006 left, p=0.009 right; precuneus p=0.012 left, p=0.013 right). MCI-Converters and AD participants showed no significant differences in either inferior parietal gyrus or precuneus cortical thickness values.

### Effect Sizes of Imaging Biomarkers

**Fig. (7). Effect sizes of the comparison between MCI-Stable and MCI-Converter groups evaluated for selected imaging biomarkers.**
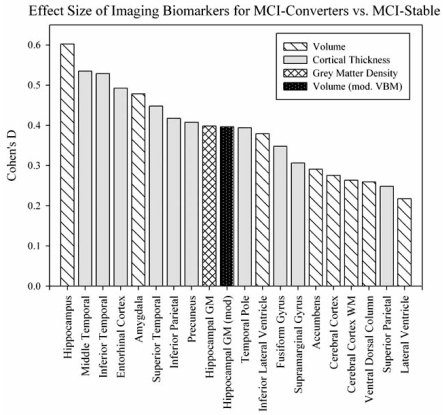
GM density, volume, and cortical thickness were extracted using VBM and automated parcellation and compared between sub-groups based on MCI to AD conversion status after one year. Effect sizes (Cohen’s d) of comparisons between MCI-Stable and MCI-Converter groups showed that imaging biomarkers from the temporal lobe, including hippocampal and amygdalar volume and cortical thickness values from the entorhinal cortex and inferior, middle, and superior temporal gyri, provided the greatest statistical difference. Age, gender, handedness, education, and ICV were included as covariates and adjusted bilateral means were used to calculate effect size.

Imaging biomarkers extracted from both VBM GM maps and automated parcellation compared between MCI-Converters and MCI-Stable participants demonstrated large effect sizes in MTL structures, as well as in temporal and parietal lobar regions (Fig. **7**). Bilateral mean hippocampal volume was found to have the highest effect size, with a Cohen’s d of 0.603. Cortical thickness values from the inferior and middle temporal gyri, as well as the entorhinal cortex, also showed strong effect sizes, with Cohen’s d values of 0.535, 0.529, and 0.493, respectively. Amygdalar volume (Cohen’s d=0.478), superior temporal cortical thickness (Cohen’s d=0.448), inferior parietal cortical thickness (Cohen’s d=0.417), precuneus cortical thickness (Cohen’s d=0.408), and hippocampal GM density (Cohen’s d=0.408) also showed high effect sizes. For illustrative purposes, imaging metrics with the 20 largest effect sizes are shown in Fig. (**7**).

## DISCUSSION

We examined baseline 1.5T T1-weighted MRI scans from 693 participants in the ADNI cohort to (1) characterize initial differences between the AD, MCI, and HC groups and (2) detect anatomic features associated with imminent conversion from MCI to probable AD within one year (MCI-Converters). We hypothesized that cross-sectional baseline differences would be consistent with the well-established progression from MTL structures to neocortical involvement, and that those individuals with MCI who are about to convert to AD will appear more similar to AD prior to conversion than those MCI patients who remain stable for at least one additional year. Publically available and widely used semi-automated image analysis methodologies (VBM in SPM5, automated parcellation in FreeSurfer) were employed to assess these hypotheses.

Several key conclusions can be drawn from the obtained results. First, the overall pattern of structural MRI changes in MCI and AD patients observed at baseline in the ADNI cohort is similar to prior findings in other, typically smaller and less intensively characterized samples [11-17, 19, 21, 25-36, 38-40, 43-46, 52, 53, 57, 73]. Second, MCI-Converters are distinguishable from individuals with MCI who will not show significant clinical progression over the next year (MCI-Stable). Third, MCI-Converters show significantly greater global and MTL-specific atrophy than MCI-Stable participants, a pattern previously reported in earlier studies with smaller samples [12, 13, 15, 18, 20, 22, 23, 31-36, 40-45, 74, 75]. Fourth, MCI-Converters show a neuroimaging profile more similar to that seen in the AD group than that of the MCI-Stable group. The MCI-Converter group demonstrated a pattern of atrophic changes nearly equivalent to those of the AD group up to a year before meeting clinical criteria for probable AD. Finally, a comparison of effect sizes for contrasts between the MCI-Converter and MCI-Stable groups on MRI metrics indicated that degree of neurodegeneration of MTL structures is the best antecedent MRI marker of imminent conversion, with decreased hippocampal volume (left more than right) being the most robust structural MRI feature.

There are several aspects to these results and analyses that warrant comment. This report is among the first, in the fully enrolled ADNI cohort, to assess group differences between AD, MCI, and controls at baseline, as well as to examine antecedent imaging predictors of future change in clinical status (i.e., conversion to probable AD, in patients with amnestic MCI). Our comparisons of the three baseline diagnostic groups using VBM are similar to previous reports using alternative methods to compare global atrophy between AD, MCI, and HC participants in the ADNI cohort [76-78]. One recent study from Hua *et al.* [77] found significant MTL atrophy in both AD and MCI subjects in the ADNI cohort using tensor-based morphometry (TBM), similar to our results using VBM. Furthermore, our results using the one year MCI to AD converter population from the ADNI cohort provided congruent results with those of Hua *et al.*, in which temporal lobe atrophy as assessed using TBM correlated with MCI to AD conversion in a subset of the ADNI MCI-Converters (n=40) [77]. A recent study using another imaging analysis technique (RAVENS) also found a similar pattern of distinctive atrophy in MCI-Converters relative to MCI-Stable participants in a sub-sample (27 MCI-Converter, 76 MCI-Stable) of the ADNI cohort, which could be used to predict MCI to AD conversion using a pattern classification technique [78]. In the present study, we were able to substantially extend the results of earlier partial cohort analyses by including the largest possible set of ADNI participants with usable data, since one year outcomes were only recently completed. Further, our multi-method approach included VBM-based analyses of GM density and volume and FreeSurfer-derived ROI analyses of volume and cortical thickness, which together provide a more detailed picture of anatomical differences between groups.

Studies employing VBM methods differ with regard to including a volume conserving step referred to as “modulation” [8-10]. Briefly, unmodulated GM maps are typically interpreted as indicating differences in GM density or concentration. By contrast, VBM performed on modulated GM maps are interpreted as local GM volume estimates. At present there is no strong consensus in the literature regarding which approach is more appropriate for a given application. Furthermore, the pathophysiological significance of differences detected by one method versus the other has not been conclusively determined. Our primary VBM analyses were performed without modulation. We then repeated the analyses with the modulation step for comparison, and found highly similar patterns of GM differences between groups (Figs. **2 & 3**, modulated data not shown). Specifically, the overall pattern of GM reduction for all patient groups (AD, MCI-Converters, MCI-Stable) compared to HC participants remained significant using both VBM methods, with the greatest differences remaining in bilateral MTL. Similarly, the pattern for MCI-Converters relative to the MCI-Stable participants was largely unaffected by analytic methodology. Analysis of GM values extracted from left, right and combined hippocampal ROIs defined in an independent cohort of healthy older adult controls [25, 58] showed similar group differences, and the effect size for MCI-Converters versus MCI-Stable participants was nearly identical (Figs. **4 & 7**, modulated data not shown). Overall, inclusion of a volume conserving modulation step in the VBM analyses had little influence on the pattern or magnitude of group differences. This may in part be related to our inclusion of intracranial volume as a covariate in all analyses. To eliminate the possibility of bias due to markedly unequal group sizes when comparing MCI-Converters and MCI-Stable participants, we repeated the main VBM analyses on four matched groups of equal size. Despite slightly attenuated power to detect group differences, the additional matched group analyses did not alter the overall pattern of results (data not shown).

The second major approach to assessing morphological changes in AD, MCI and controls entailed examining Free-Surfer parcellation derived ROIs, selected on the basis of their status as important regions for AD pathology. Group differences were evaluated for left, right and combined hippocampal volume and GM density, additional MTL ROIs, and regional cortical thickness estimates. Significant differences between groups were found in hippocampal, amygdalar, and other MTL regions, as well as widespread neocortical regions. These results are consistent with prior ROI and VOI studies in AD and MCI, in which hippocampal volumes [17, 25, 31-36, 38-40, 44, 48, 52, 53, 79], hippocampal GM density [19, 24, 25], and other regions [19, 31-35, 38, 39, 43-47, 50, 51, 74, 75], were found to be significantly decreased relative to HC. As in our VBM results, ROI measures indicated that participants who convert from MCI to AD within one year show significant atrophy relative to MCI participants who remain clinically stable, and also have a generally equivalent degree of atrophy to AD participants. Decreased hippocampal GM density and volume, amygdalar volume, and cortical thickness in entorhinal cortex, inferior, middle, and superior temporal gyri, inferior parietal gyrus, and precuneus reflect the antecedent structural characteristics of MCI-Converters compared to individuals with MCI who remained clinically stable for at least a year. Similar to the global atrophy detected using VBM, local measures of volume and cortical thickness detected significant degeneration in MCI-Converters up to one year prior to the point at which they meet clinical criteria for an AD diagnosis, suggesting an accelerated rate of neuropathological changes in these individuals which is not well captured by the MCI diagnosis alone. Furthermore, these results, obtained from the largest group assessment of one year conversion from MCI to AD to date, extend the findings of previous smaller studies which have reported local atrophy in MCI to AD converters using measures of hippocampal, amygdalar, entorhinal cortex, and other MTL volume estimates [12, 13, 15, 18, 20, 23, 31-34, 36, 37, 40, 42-45, 50, 74, 75, 80].

Taken together, the present findings support the use of structural MRI as a biomarker for assessing prodromal and early AD related changes. An important implication of the analyses performed in this report is that although many regions and measurements are sensitive to early AD pathology, MRI markers have differential sensitivities for detection of those individuals who are at greatest risk of short-term progression to probable AD. The MRI measures with the largest effect sizes (far left, Fig. **7**) for MCI-Converters versus MCI-Stable contrasts appear to be important biomarker candidates for prediction of MCI to AD conversion. Previous studies have investigated the use of MTL density and volume in the prediction of MCI to AD conversion, with some reports finding significantly greater sensitivity and specificity achieved by adding imaging biomarkers to clinical test prediction algorithms, while others suggested minimal utility of including additional imaging variables [31-34, 37, 42-45, 47, 48, 51, 74, 75, 79, 81]. However, the majority of these studies included modest participant pools and manually drawn ROIs. The time-consuming nature of manual ROI tracing limits the utility of these endpoints as biomarkers in studies with large numbers of participants, as well as in routine clinical settings. Automated or semi-automated extraction of volume and cortical thickness values from ROIs in the MTL requires minimal manual intervention. The largely automated nature and wide availability and use of this and other methods (e.g., [53, 82]) in assessing local and global atrophy will facilitate incorporation of these measures as key variables in pharmacological efficacy and neuroprotection trials.

A limitation of the present report is the inclusion of only baseline scans in characterizing anatomic changes. Additional information, including changes in imaging measures over time and rate of atrophy, has been shown to be useful in assessing and accurately predicting rapid conversion [41, 42]. As a cross-sectional assessment of structural neuroimaging measures, the present study does not capture the dynamic processes associated with MCI to AD conversion. Future studies assessing multiple timepoints, including two and three year MCI to AD conversion patterns, will be needed to determine the diagnostic and predictive value of dynamic measures of global and local atrophy. Furthermore, the current participant pool includes 182 participants diagnosed with MCI at baseline who were on AD-indicated medications during the first year of the study. Pharmacological treatments, such as AChE inhibitors and memantine, have been shown to reduce or delay MCI to AD conversion [83-87]. The impact of medications was not assessed in the current study. Future studies should focus on including this variable in predicting and assessing conversion from MCI to AD. Another limitation of this report is the inclusion of only structural imaging. It is possible that FDG PET, obtained on approximately half of the ADNI cohort, could enhance the detection and characterization of antecedent changes alone or in combination with MRI and other measures. Targeted molecular PET imaging for amyloid deposition with [11C]PiB is also being investigated in a smaller add-on study in the ADNI cohort [88]. Future studies will undoubtedly clarify the contribution of FDG and PiB PET to understanding early changes and predicting clinical trajectory. Finally, the role of genetic factors was only considered to a limited degree in the present study by controlling for APOE genotype where appropriate. A genome wide association study employing a high density microarray with over 620,000 single nucleotide polymorphisms is underway by the ADNI Genetics Working Group and these forthcoming results will permit inclusion of data on individual differences in important biological pathways in predictive models.

In summary, a major goal of ADNI is to identify imaging biomarkers that could be used for early detection and prediction of longitudinal changes in MCI and AD. Two semi-automated, widely used and publically available image analysis methods (VBM, automated parcellation) revealed significant global and local atrophy in AD and MCI patients in a large cohort from the ADNI sample at baseline relative to HC. These techniques were also successful at detecting differences at baseline between participants who would convert from MCI to AD within one year and those who would remain stable with an MCI diagnosis for at least one year. The results of these analyses suggest that VBM and automated parcellation are useful tools for characterization of atrophy in MCI and AD and prediction of disease course. Employed with repeated scans for longitudinal monitoring of brain degeneration, these methods will be useful for clinical trials in MCI and AD. With further refinement, MRI coupled with advanced image analysis approaches appears to have potential for individualized prediction of risk of progression and enhancement of clinical trials by including those at greatest risk of conversion.

## FUNDING

Data collection and sharing was funded by the Alzheimer’s Disease Neuroimaging Initiative (ADNI; Principal Investigator: Michael Weiner; NIH grant U01 AG024904). ADNI is funded by the National Institute on Aging (NIA), the National Institute of Biomedical Imaging and Bioengineering (NIBIB), and through generous contribution from the following: Pfizer Inc., Wyeth Research, Bristol-Myers Squibb, Eli Lilly and Company, GlaxoSmithKline, Merck & Co. Inc., AstraZeneca AB, Novartis Pharmaceuticals Corporation, the Alzheimer’s Association, Eisai Global Clinical Development, Elan Corporation plc, Forest Laboratories, and the Institute for the Study of Aging, with parti-cipation by the U.S. Food and Drug Administration. Industry partnerships are coordinated through the Foundation for the National Institutes of Health. The grantee organization is the Northern California Institute for Research and Education, and the study is coordinated by the Alzheimer’s Disease Cooperative Study at the University of California, San Diego. ADNI data are disseminated by the Laboratory of Neuro Imaging at the University of California, Los Angeles.

Data analysis was supported in part by the following grants from the National Institutes of Health: NIA R01 AG19771 to AJS and P30 AG10133 to Bernardino Ghetti, MD and NIBIB R03 EB008674 to LS, and by the Indiana Economic Development Corporation (IEDC #87884 to AJS).

## Figures and Tables

**Fig. (3). Group comparisons of patient groups based on baseline diagnosis and one year conversion status using a one-way ANOVA of GM density maps.**
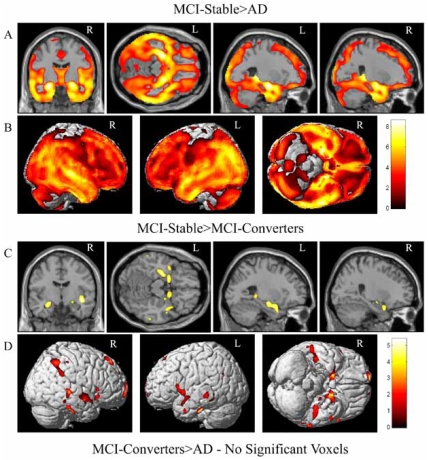
Selected slices (**A**) and surface renderings (**B**) of regions where MCI-Stable>AD. Selected slices (**C**) and surface renderings (**D**) of regions where MCI-Stable>MCI-Converters. No significant voxels were found in the comparison between MCI-Converters and AD participants. All comparisons are displayed at a threshold of p<0.005 (FDR), k=27. Using a more lenient statistical threshold, differences were apparent in the posterior parietal and occipital lobes (data not shown). Age, gender, years of education, handedness and ICV were included as covariates in all comparisons. Selected sections for (**A**) and (**C**) include left to right MNI coordinates: (0, -9, 0, coronal), (0, -23, -16, axial), (-26, -10, -15, sagittal), and (26, -10, -15, sagittal).

**Table 1:** ADNI Participants at Baseline (Adjusted Mean (SE))

	AD	MCI-Converters	MCI-Stable	HC	p-value	Pairwise Comparisons (at p<0.01)
	(n=148)	(n=62)	(n=277)	(n=206)		
Age (yrs.)	75.4 (0.6)	74.3 (0.9)	75.1 (0.4)	76.0 (0.5)	NS	No pairs significant
Gender (M, F)	77, 71	36, 26	178, 99	107, 99	0.02	MCI-S>HC
Education (yrs.)	14.8 (0.2)	15.2 (0.4)	15.8 (0.2)	16.1 (0.2)	p<0.001	HC, MCI-S>AD
Handedness (R, L)	141, 7	57, 5	253, 24	189, 17	NS	No pairs significant
% English Speaking	98.7%	98.4%	97.5%	99.0%	NS	No pairs significant
% ApoE ε4 Positive (1 or 2 alleles)	65.5%	59.7%	53.1%	27.2%	p<0.001	AD, MCI-C, MCI-S>HC

MMSE^e^	23.5 (0.1)	26.7 (0.2)	27.1 (0.1)	29.1 (0.1)	p<0.001	HC>all^f^; MCI-S, MCI-C>AD
Global CDR^e^	0.75 (0.01)	0.50 (0.02)	0.50 (0.01)	0.00 (0.01)	p<0.001	AD>all^g^; MCI-S, MCI-C>HC
CDR – Sum of Boxes^e^	4.3 (0.1)	1.9 (0.1)	1.5 (0.1)	0.3 (0.7)	p<0.001	AD>all^g^; MCI-S, MCI-C>HC
FAQ^a^,^e^	13.0 (0.4)	6.4 (0.5)	3.2 (0.3)	0.1 (0.3)	p<0.001	All pairs significant

Geriatric Depression Scale^e^	1.6 (0.1)	1.3 (0.2)	1.6 (0.1)	0.8 (0.1)	p<0.001	AD, MCI-S>HC
NPI-Q^e^	3.5 (0.2)	2.2 (0.3)	1.7 (0.2)	0.4 (0.2)	p<0.001	AD>all^g^; MCI-S, MCI-C>HC
Modified Hachinski^e^	0.64 (0.06)	0.63 (0.09)	0.65 (0.04)	0.57 (0.05)	NS	No pairs significant

RAVLT (1-5)^b^,^e^	23.5 (0.7)	26.2 (1.1)	31.9 (0.5)	42.5 (0.6)	p<0.001	HC>all^f^; MCI-S>MCI-C, AD
RAVLT 30min Recall^c^,^e^	0.8 (0.3)	1.2 (0.4)	3.1 (0.2)	7.5 (0.2)	p<0.001	HC>all^f^; MCI-S>MCI-C, AD
RAVLT 30min Recognition^c^,^e^	7.4 (0.3)	8.1 (0.4)	10.0 (0.2)	13.0 (0.2)	p<0.001	HC>all^f^; MCI-S>MCI-C, AD
Boston Naming Test^d^,^e^	22.8 (0.3)	24.4 (0.5)	25.5 (0.2)	27.9 (0.3)	p<0.001	HC>all^f^; MCI-S>AD
Fluency - Animals^e^	12.7 (0.4)	14.3 (0.6)	16.2 (0.3)	20.1 (0.3)	p<0.001	HC>all^f^; MCI-S>AD
Fluency - Vegetables^e^	7.8 (0.3)	9.3 (0.4)	11.2 (0.2)	14.7 (0.2)	p<0.001	HC>all^f^; MCI-S>MCI-C, AD

Total Intracranial Volume (ICV)^e^	1607159.5 (14004.6)	1598192.9 (21470.1)	1597844.8 (10181.0)	1576429.8 (11855.6)	p<0.001	No pairs significant

a 3 MCI-Stable participants removed due to incomplete scores

b 7 participants removed due to incomplete scores (3 AD, 4 HC)

c 1 HC participant removed due to an incomplete score

d 3 participants removed due to incomplete scores (1 AD, 1 MCI-Stable, 1 HC)

e Covaried for age, education, gender, and handedness

f HC>all is HC>MCI-S, MCI-C, AD

g AD>all is AD>MCI-S, MCI-C, HC (Note: greater scores on these measures (CDR, FAQ, GDS, NPI-Q, Modified Hachinski) signify more impairment)

## ABBREVIATIONS

MRI: = Magnetic resonance imaging
ADNI: = Alzheimer’s Disease Neuroimaging Initiative
AD: = Alzheimer’s Disease
MCI: = Mild Cognitive Impairment
HC: = Healthy elderly controls
MTL: = medial temporal lobe
GM: = Grey matter
MP-RAGE: = Magnetization prepared rapid acquisition gradient echo
SPM: = Statistical parametric mapping
VBM: = Voxel-based morphometry
ROI: = Region of interest
VOI: = Volume of interest
FDR: = False discovery rate
ApoE: = Apolipoprotein E
